# The Safe Administration of Remdesivir in a Patient with Acute Kidney Injury Requiring Hemodialysis

**DOI:** 10.1155/2020/8811798

**Published:** 2020-12-27

**Authors:** Vincent Peyko, Helena Ladd, Anthony Cutrona

**Affiliations:** ^1^Mercy Health–St Elizabeth Boardman Hospital, Department of Pharmacy, 8401 Market St., Boardman, Youngstown, OH 44512, USA; ^2^NEO Infectious Diseases Associates, 540 Parmalee Ave., Ste. 610, Youngstown, OH 44510, USA

## Abstract

Remdesivir is a nucleoside analog prodrug with broad-spectrum antiviral activity, including against coronaviruses. This has prioritized the inclusion of remdesivir in coronavirus disease 2019 (COVID-19) clinical trials. The United States Food and Drug Administration has granted emergency use authorization for remdesivir. This emergency use authorization does not recommend the use of remdesivir in patients with estimated glomerular filtration rate (eGFR) less than 30 mL/min unless the benefits outweigh the risks. To date, there are no studies and scant information in the literature evaluating remdesivir utilization in patients with eGFR less than 30 mL/min or receiving hemodialysis. With little utilization data for patients with acute or chronic kidney injury, remdesivir may not be considered, leaving this patient population without the opportunity of a potentially beneficial treatment option. We present a case of one patient with eGFR less than 30 mL/min that required hemodialysis in which remdesivir was safely initiated, with therapy completed without any serious adverse events.

## 1. Introduction

Remdesivir is a nucleoside analog prodrug initially developed in response to Ebola [1]. The nucleoside core, GS 441524, is the primary circulating metabolite [2]. Remdesivir inhibits viral replication by competing with endogenous nucleotides for incorporation into replicating viral RNA through the RNA-dependent RNA polymerase (RdRp) [1]. An RdRp nonstructural protein, nsp12, is highly conserved amongst coronaviruses making it an attractive target for broad-spectrum antiviral drugs [1]. This broad-spectrum activity against coronaviruses has prioritized the inclusion of remdesivir in coronavirus disease 2019 (COVID-19) clinical trials [1]. Utilization of remdesivir to treat COVID-19 in the United States was first reported in late January 2020 [3]. Currently, 44 studies are registered at clinicaltrials.gov to evaluate utilization of remdesivir in COVID-19 [4]. Comparisons of remdesivir to placebo have had mixed results, although the largest clinical trial (ACTT-1) demonstrated that remdesivir reduced time to recovery in patients hospitalized with COVID-19 versus placebo [5, 6]. These results were promising enough for the FDA to issue an emergency use authorization (EUA) for remdesivir [7]. The EUA states that remdesivir is not recommended in patients with estimated glomerular filtration rate (eGFR) less than 30 mL/min unless the potential benefits outweigh the risk [7]. Without utilization data on patients with acute or chronic kidney injury, remdesivir may not be considered, leaving this patient population without the opportunity of a potentially beneficial treatment option [8]. At this time, data are scant in the literature describing utilization of remdesivir in a patient with eGFR less than 30 mL/min or on hemodialysis. We present a case of a patient initiated on remdesivir with eGFR less than 30 mL/min that required hemodialysis as the result of acute kidney injury.

## 2. Case Report

A 67-year-old male presented to the emergency department (ED) with altered mental status and difficulty breathing following recent COVID-19 exposure. The patient had experienced progressing shortness of breath and confusion for several days prior to arriving at the hospital. Past medical history includes obesity, chronic obstructive pulmonary disease, obstructive sleep apnea, hypertension, current smoker, and alcohol abuse. Oxygen saturation on presentation was 36% on room air, improving to 78% on 15 L non-rebreather mask leading to intubation. In the ED, the patient was febrile with a core temperature of 38.6°C, body mass index was 40.08 kg/m^2^, heart rate was 116 beats/min, respiratory rate was 16 breaths/min, blood pressure was 182/73 mmHg, and SARS-CoV-2 nucleic acid amplification test (NAAT) (Abbot® ID NOW COVID-19) was positive. Initial laboratory values included serum creatinine of 5.1 mg/dL (baseline of 1.2 mg/dL), C-reactive protein 32.3 mg/dL, lactate dehydrogenase 759 U/L, pro-BNP 311 pg/mL, ferritin 561 ng/mL, D-dimer 1630 ng/mL, procalcitonin 2.68 ng/mL, and fibrinogen >700 mg/dL. Arterial blood gas results included pH of 7.331, PaCO_2_ 40.9 mmHg, HCO_3_ 44.3 mmol/L, and PaO_2_ 21.1 mmHg, with oxygen saturation 74.2%. Azithromycin and ceftriaxone were initiated for community-acquired pneumonia empiric treatment.

The patient was admitted to the intensive care unit (ICU) mechanically ventilated with acute kidney injury (AKI) in the setting of COVID-19. Due to the extent of his illness, infectious diseases, nephrology, and pharmacy teams discussed therapy for COVID-19 treatment in the setting of AKI and the decision was to initiate remdesivir the evening of admission. Tocilizumab 400 mg intravenously (IV) was also given once on the evening of admission due to elevated inflammatory markers with presumed cytokine release syndrome. Dexamethasone 6 mg IV once daily was initiated the following morning, and the patient had a temporary hemodialysis catheter placed with sustained low-efficiency dialysis (SLED) initiated.

Within 24 hours of admission, the patient remained in the ICU mechanically ventilated in prone position, sedated on propofol and fentanyl titrated continuous infusions, paralyzed on cisatracurium 2 mcg/kg/min continuous infusion, and hemodynamically supported with normal saline and titrated phenylephrine infusion. Heparin 5,000 units subcutaneously every 8 hours was initiated for thromboembolic prophylaxis. Vitamin C 1500 mg IV every 6 hours, ergocalciferol 4,000 units per nasogastric tube once daily, thiamine 100 mg IV every 12 hours, and piperacillin/tazobactam 3.375 g IV every 8 hours were all initiated within 24 hours of admission. Renal replacement therapy was progressed to intermittent hemodialysis on day 2.

On day 3, D-dimer increased to 3425 ng/mL and the patient was switched to full-dose anticoagulation via heparin continuous infusion with a goal aPTT of 50–80 seconds. Phenylephrine was weaned off and changed to midodrine 20 mg by mouth every 8 hours. Piperacillin/tazobactam was discontinued after 5 days of treating tracheitis as cultures were negative for growth.

After day 5, the course of remdesivir 200 mg IV once on day 1 followed by remdesivir 100 mg IV once daily for the next 4 days was completed. On day 6, heparin infusion was stopped due to positive fecal occult blood test with sequential compression devices applied for thromboembolic prophylaxis. On day 7, duplex ultrasonography demonstrated bilateral lower extremity deep vein thrombosis (DVT) with heparin 5,000 units subcutaneously every 8 hours reinitiated in the setting of hemoccult-positive stools and the patient not being a candidate for inferior vena cava filter due to COVID-19.

The patient was initially managed in the ICU while mechanically ventilated. The patient continued to slowly improve daily, demonstrated by ventilation settings. He was ultimately extubated and transferred out of the ICU on day 15. After extubation, he remained on nasal cannula with continual improvement in pulse oximetry and reduced oxygen requirements.

The patient had consecutive negative SARS-CoV-2 NAATs (LabCorp Laboratories, Covance Central Laboratory) on days 16 and 19 after admission.

On the day of his discharge, the patient required 1 liter of supplemental oxygen via nasal cannula with a goal pulse oximetry reading greater than 90% with further weaning as the patient was not on any oxygen prior to illness.

**Figure 1:**
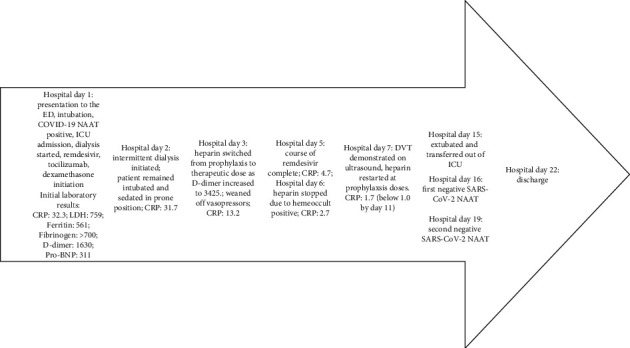
Timeline of hospital course. CRP: C-reactive protein (mg/dL); LDH: lactate dehydrogenase (U/L); Pro-BNP: pro-beta natriuretic protein (pg/mL). Units: D-dimer: ng/mL; ferritin: ng/mL; fibrinogen: mg/dL.

After 22 days of hospitalization, the patient was discharged to a skilled nursing facility (Figure 1). The patient remained oliguric for the length of his admission, requiring intermittent hemodialysis. The plan was to continue thrice weekly intermittent hemodialysis and full-dose anticoagulation on discharge.

During the entire length of stay, there were no adverse events or drug interactions related to the medical management of this patient.

## 3. Discussion

The safety and efficacy of remdesivir has not been extensively studied in patients with severe kidney dysfunction or end-stage renal disease [7]. Severe COVID-19 may lead to acute kidney injury in 20–40% of critically ill patients [8]. This lack of safety and efficacy data has led to the EUA recommending the provider to weigh the risks versus benefits in utilizing remdesivir in this patient population [7].

Remdesivir is considered to pose a low potential for mitochondrial toxicity because it is only a weak inhibitor of mammalian DNA and RNA polymerases [8]. Other nucleotide/nucleoside antivirals can lead to mitochondrial toxicity in the renal tubules but occurs with prolonged exposure; thus, toxicity from 5- to 10-day courses of therapy would be considered rare [8]. Renal adverse events were not reported when remdesivir was studied in Ebola patients [9]. In the largest, double-blind, randomized placebo-controlled trial of remdesivir, there was not increased renal toxicity in those receiving remdesivir versus placebo [5]. A case series of 46 patients with AKI or chronic kidney disease (CKD) that included 16 patients with end-stage renal disease (ESRD) demonstrated no discontinuation of therapy due to side effects [10]. A case series of 18 patients demonstrated a lack of serious safety concerns in patients on renal replacement therapy or with CrCl <30 mL/min, but did not describe drug concentrations [11].

Less than 10% of remdesivir is excreted renally [1]. However, 49% of a remdesivir metabolite (GS-441524) has been recovered in urine [1]. Thus, theoretically, accumulation of this metabolite may occur in patients with impaired renal function although untoward effects remain unknown [1]. An evaluation of plasma concentrations in 3 patients on hemodialysis receiving remdesivir determined increased remdesivir plasma-half life from 1 to 2 hours, when compared to healthy volunteers, which would maintain therapeutic remdesivir plasma levels [2]. As remdesivir concentration falls, up to a 10-fold increase in the metabolite GS-441524 was observed, presumably due to conversion of active drug to metabolite [2]. Accumulation of GS-441524 is thought to occur due to impaired renal elimination; however, no adverse effects were attributed to metabolite accumulation [2].

We initiated remdesivir therapy prior to placement of dialysis catheter while eGFR was 14 mL/min, below the threshold of an eGFR of 30 mL/min, because we felt that the potential benefits outweighed the risks in accordance with the FDA EUA [7]. The plan was also to initiate dialysis the following day, which would also remove sulfobutylether-beta-cyclodextran (SBECD).

SBECD is a solubility-enhancing agent to improve the solubility of remdesivir [1, 8]. SBECD is predominantly excreted through glomerular filtration. Thus, accumulation and theoretical toxicity is hypothesized in patients with impaired renal function [1, 8]. Animal studies in rats and dogs have demonstrated mild kidney toxicity in doses approximately 50-fold greater than SBECD doses typically administered in humans [12]. Each 100 mg vial of remdesivir lyophilized powder contains 3 grams of SBECD, while 100 mg vials of remdesivir solution contains 6 grams of SBECD, which would be far below SBECD recommended safety thresholds of 250 mg/kg/day [8]. Voriconazole is an azole antifungal medication whose IV formulation contains SBECD, with much of what is known about the pharmacokinetics and clinical effects of SBECD in renal failure extrapolated from voriconazole data [8]. Hemodialysis significantly removes SBECD and reduces SBECD levels [13]. While SBECD may accumulate in patients with impaired renal function, there is no evidence this accumulation leads to any adverse events [8, 13, 14]. Respiratory failure, increased transaminases, thrombocytopenia, hypokalemia, hyperkalemia, and increased bilirubin are all additional adverse events reported with remdesivir use [1].

The efficacy of remdesivir remains unclear. To date, there are two published randomized, double-blind, placebo-controlled studies evaluating remdesivir [5, 6]. A Chinese study, conducted between February 6 and March 12, 2020, evaluated 237 patients randomized to receive 10 days of remdesivir or placebo which demonstrated no significant difference in clinical benefits between groups [6]. However, there was a numerically faster time to clinical improvement with remdesivir versus placebo [6]. A multinational study (ACTT-1) conducted between February 21 and April 19, 2020, evaluated 1059 patients randomized to receive 10 days of remdesivir or placebo and demonstrated that remdesivir was superior to placebo in shortening time to recovery in adults hospitalized with COVID-19 [5]. Patients receiving remdesivir had a median time to recovery of 11 days versus 15 days in patients receiving placebo (*p* < 0.001) [5]. Our patient only received 5 days of remdesivir therapy due to similarities demonstrated in clinical status in 397 patients that received 5 versus 10 days of remdesivir [15].

There was no significant difference in adverse events between patients given remdesivir versus placebo in both published randomized, double-blind, placebo-controlled studies evaluating remdesivir [1, 5, 6]. No adverse events reported in the manuscript or supplemental index of ACTT-1, including the most commonly cited of anemia, decreased hemoglobin, acute kidney injury, pyrexia, hyperglycemia, or increased aminotransferase levels, amongst others occurred in our patient [5, 16]. No serious adverse events reported in Wang et al.'s study could be attributed to remdesivir in our patient [6]. Our patient did experience elevated D-dimer and DVT, which were considered adverse events in Wang et al.'s study [6]. However, D-dimer was elevated at baseline and DVT was diagnosed two days after remdesivir completion. Hypercoagulability is commonly the result of COVID-19, and our patient was not receiving thromboembolic chemoprophylaxis at the time of DVT diagnosis. Thus, we do not attribute this to be the result of remdesivir administration [17–20]. Particularly considering, there was no difference in the occurrence of increased D-dimer or DVT in patients receiving remdesivir versus placebo [6]. Thus, we could attribute no adverse events as the result of remdesivir administration in our patient.

Other factors may have affected our patient's outcome. The patient received two other medications utilized in the potential treatment of COVID-19. Dexamethasone 6 mg IV once daily for 10 days was administered to this patient. The Randomized Evaluation of COVID-19 Therapy (RECOVERY) trial demonstrated the ability of dexamethasone 6 mg once daily to reduce 28-day mortality in hospitalized patients with COVID-19 on oxygen or mechanical ventilation (odds ratio, 0.66; 95% CI, 0.53–0.82; *p* < 0.001) [21].

The patient also received tocilizumab 400 mg IV once. A randomized, placebo control study of 389 patients receiving tocilizumab vs. standard care demonstrated the efficacy and safety of tocilizumab compared to placebo in reducing the likelihood of progression to requiring mechanical ventilation or death in nonventilated patients [22]. A retrospective study of 5,776 patients in which 454 patients received the combination of tocilizumab and steroids demonstrated improved survival versus patients that received standard care (hazard ratio (HR), 0.44; 95% confidence interval (CI), 0.35–0.55; *p* < 0.0001) or standard care plus steroids (HR, 0.66; 95% CI, 0.53–0.83; *p*=0.004) [23]. However, a randomized, placebo control study of 452 patients receiving tocilizumab vs. standard care did not improve clinical status or mortality of tocilizumab compared to placebo in this patient population which included those mechanically ventilated [24]. Our patient was mechanically ventilated at tocilizumab initiation, and thus, it is unclear whether tocilizumab may have helped our patient. There are also other studies suggesting the lack of effect of tocilizumab; however, the effect of concomitant steroids was not evaluated [25, 26]. These conflicting results suggest the combination of tocilizumab and steroids may or may not have affected our patient's outcome. Furthermore, we cannot quantify the effect of supportive care with this patient.

Whether or not remdesivir, dexamethasone, tocilizumab, supportive care, or other factors, alone or as a combination, led to the successful discharge of this patient remains unknown. However, we did not observe any drug-related adverse effects due to the administration of remdesivir [5, 6].

Outside of COVID-19, reduced length of hospital stay may decrease 30-day readmission rates and mortality [27, 28]. While further study is needed to determine if decreased time to recovery leads to decreased length of stay with COVID-19, our experience shows this to be accurate. The current literature does demonstrate that remdesivir may reduce the median time to recovery [5]. Our case presented demonstrates no adverse events related to the administration of remdesivir in our patient with an eGFR less than 30 mL/min that required hemodialysis while receiving remdesivir. As such, while further study is needed, we recommend that remdesivir therapy not be withheld due to reduced eGFR or hemodialysis alone.

## Data Availability

No data were used to support this study.
